# Monthly food insecurity assessment in rural mkushi district, Zambia: a longitudinal analysis

**DOI:** 10.1186/s12889-017-4176-0

**Published:** 2017-03-16

**Authors:** Muzi Na, Bess L. Caswell, Sameera A. Talegawkar, Amanda Palmer

**Affiliations:** 1 Center for Human Nutrition, Department of International Health, Johns Hopkins Bloomberg School of Public Health, Baltimore, MD USA; 2 Department of Exercise and Nutrition Sciences, Milken Institute School of Public Health, The George Washington University, Washington, DC USA

**Keywords:** Food insecurity, Perception-based scale, Longitudinal monitoring, Zambia

## Abstract

**Background:**

Perception-based scales are widely used for household food insecurity (HFI) assessment but were only recently added in national surveys. The frequency of assessments needed to characterize dynamics in HFI over time is largely unknown. The study aims to examine longitudinal changes in monthly reported HFI at both population- and household-level.

**Methods:**

A total of 157 households in rural Mkushi District whose children were enrolled in the non-intervened arm of an efficacy trial of biofortified maize were included in the analysis. HFI was assessed by a validated 8-item perception-based Likert scale on a monthly basis from October 2012 to March 2013 (6 visits), characterizing mostly the lean season. An HFI index was created by summing scores over the Likert scale, with a possible range of 0–32. The Wilcoxon matched signed-ranks test was used to compare distribution of HFI index between visits. A random effect model was fit to quantify the sources of variance in indices at household level.

**Results:**

The median [IQR] HFI index was 4.5 [2, 8], 5 [1, 8], 4 [1, 7], 4 [1, 6], 3 [1, 7] and 4 [1, 6] at the six monthly visits, respectively. HFI index was significantly higher in visit 1 and 2 than visit 3–6 and on average the index decreased by 0.25 points per visit. Within- and between-household variance in the index were 10.6 and 8.8, respectively.

**Conclusions:**

The small change in mean monthly HFI index over a single lean season indicated that a seasonal HFI measure may be sufficient for monitoring purposes at population level. Yet, higher variation within households suggests that repeated assessments may be required to avoid risk of misclassification at household level and to target households with the greatest risk of food insecurity.

## Background

Despite gains in reducing hunger over the past 15 years, 791 million people world-wide (1 in 9 individuals) are still classified as chronically hungry based on insufficient food to meet their energy requirements [1]. The proportion of households considered food insecure is the highest in Sub-Saharan Africa, where almost one in four individuals have inadequate dietary energy intakes [1]. Existing monitoring efforts are primarily carried out on an annual or seasonal basis and aggregated at the regional or national level [2]. However, the global food crisis in 2008 has prompted calls for better longitudinal monitoring of food insecurity with greater focus on vulnerable areas.

Perception-based food insecurity questionnaires are designed to capture a set of common experiences and behaviors that reflect the progressive stages of food access insecurity [3]. Respondents are asked about psychological concerns, compromises on food quality, reduction in food quantity, and other socially acceptable means of coping with food stress at household- or individual-levels. Though subjective reporting of household or personal experience introduces some risk of biased responses and misclassification [4], perception-based measures frequently covary in the expected direction with objective food insecurity proxies, such as household wealth [5], income [6], food expenditure [7], dietary diversity [8, 9], and nutritional outcomes [10, 11].

Despite the utility of perception-based indicators of food insecurity and their increasing use in monitoring and program evaluation, little is known about the frequency of assessments required to adequately quantify longitudinal changes in HFI. Presumably, monthly assessments would provide additional information to identify at-risk populations and to target interventions to those with the greatest need. Research carried out in rural Bangladesh indicated that two or three repeated food insecurity assessments can reflect a static HFI status, with the degree of food insecurity remaining almost unchanged over a few months [12] to approximately one year [13]. However, assessments in Sub-Saharan Africa conducted in lean and harvest months suggest seasonal variation in reported food insecurity status [14, 15].

To address this gap in knowledge, our aims were to examine changes in monthly household food insecurity (HFI) status at the population and household level over a six-month period. We hypothesized that the reported HFI would be the lowest in the late post-harvest season (October-January) and highest at the peak of the lean season (~March-April).

## Methods

### Subjects and data collection

This study was carried out in Mkushi District—a rural agricultural area located in Central Province, Zambia—from September 2012 to March 2013 [16]. We enrolled 1,226 children aged 4–8 years, not yet attending school, from 907 households in a cluster-randomized efficacy trial of provitamin A carotenoid biofortified maize. A total of 64 clusters were randomized to a six-month feeding intervention with biofortified (*n* = 25 clusters) or conventional (*n* = 25 clusters) maize meal, or a parallel non-intervened arm (*n* = 14 clusters). Households in the non-intervened arm received an equivalent food package after the six-month field period. To avoid potential influence of the meal intervention on perceived food insecurity, we have only used data from the non-intervened arm in this analysis. In September 2012, trained field interviewers visited consenting households to collect baseline data on demographics, socio-economic status, and HFI. Households were revisited in the subsequent six months (October 2012 to March 2013) to collect repeated information on food insecurity status. In this part of Zambia, the rainy season starts in November or December and lasts until April. The main planting of maize occurs early in the rainy season. The lean season is from January until March or early April, when the main harvest season starts. The climate is cool and dry from May to August and hot and dry from September to November. Food is readily available from the harvest season in the cool-dry season and becomes limiting in the hot-dry season, which is often referred as post-harvest season. The field period of this study can be characterized as the lean season.

### Household food insecurity (HFI) assessment

We adapted the Food Access Survey Tool (FAST) to measure HFI by changing the staple crop consumption questions from rice to maize. FAST is a 9-item Likert scale, which was developed [17] and validated [5] to assess HFI in Bangladesh. Respondents are asked about their experiences or concerns regarding food acquisition (worrying about food; purchasing maize often; running out of food), reduction in food quality and quantity (eating square meals; consuming other grains when maize is preferred; eating less food; skipping meals), and other coping behaviors used to buffer food shortages (taking food on credit from shops; borrowing food from relatives or neighbours). Respondents were asked to recall the frequency that they experienced each of the nine situations over the past six months (baseline) or the past one month (six monthly visits) on the same Likert scale: 0 = never;1 = rarely; 2 = sometimes; 3 = often or 4 = mostly. We used baseline data to assess the internal validity of the FAST scale [18], including examining item fitness within one food insecurity dimension and comparing estimated item severity against a theoretical expectation. Based on that analysis, we removed the item regarding frequency of eating “three square meals” from the summed score because it violates the unidimensional assumption. Given differing recall periods, we excluded the baseline data from the present analysis.

### Statistical analysis

Following a previously published method [18], we created an HFI index by tallying the eight internally validated items for each visit (i.e., excluding the “three square meals” item). A zero index indicates that a household had never experienced any of the measured situations, indicating a food-secure status. The HFI index increases with greater food insecurity. Visits with missing information on any of the eight items were assigned a missing HFI index. To understand potential selection bias, we compared baseline characteristics between households included and excluded from the analysis. For each visit, we further assessed differences between included and excluded households in terms of HFI indices at the prior visit using the Wilcoxon-Mann–Whitney test.

To explore HFI status at the population level, distributions of HFI indices by visit were first plotted in box graphs at each of the six time points. Because the HFI indices were panel data within households and distributions of this index are generally positively skewed, the Wilcoxon matched signed-ranks test was used to examine the differences in HFI distribution between visits in all possible pairs. To visualize the intra-household variance in monthly reported food insecurity within the same household and inter-household variance between different households, we plotted the mean and 95% confidence intervals (95%CI) of HFI indices under the t distribution by household. We created three HFI groups based on the mean HFI index across six visits to define households with no-mild (mean HFI < 5), moderate (mean HFI > =5 and <10) and severe (mean HFI > =10) HFI. We plotted the distribution of within-household standard deviations across the three groups to identify any potential trend of correlation between the household mean and with-household variability of the HFI index. Kendall's pairwise correlation coefficients, the statistics to measure ranked associations, were calculated among HFI indices between paired visits. The Tau-b coefficients were computed to adjust for tied values presented in HFI indices between two households at two visits (e.g. HFI indices were the same for household i and j at visit 1 and at visit 2). We then fit a random intercept model to quantify the sources of variance in HFI indices as a linear function of visit, clustered at household level:

$$ H F{I}_{i j}={U}_i+{\beta}_0+{\beta}_1 Visi{t}_j+{\varepsilon}_{i j}, $$

where HFI_ij_ is the HFI index of household i at visit j. U_i_ was the household-level random intercept following a normal distribution N(0, τ^2^). τ^2^ represented the variance between households. β_0_ was the baseline HFI index. β_1_ was the mean change of HFI index per visit. ε_ij_ was the error term following a normal distribution N(0, σ^2^). σ^2^ represented within-household variance of HFI index. The interclass correlation coefficient, ρ, was calculated as τ^2^/(τ^2^ + σ^2^) and interpreted as the proportion of total variance that can be explained by between-household variance observed.

Stata/SE 13.1 (StataCorp, College Station, TX) was used to conduct the present analyses. A p-value less than 0.05 was considered statistically significant.

## Results

**Fig. 1:**
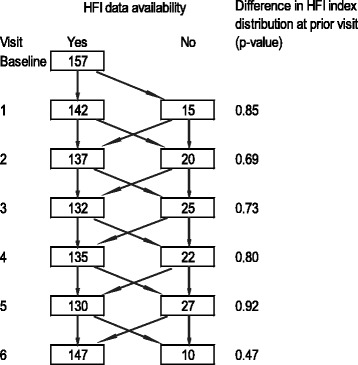
Distribution of included and excluded households at each visit. *P*-values are calculated by Wilcoxon-Mann–Whitney test. HFI, household food insecurity

We recruited eligible children (*n* = 202) from 157 households into the trial’s non-intervened arm. Panel food insecurity data was missing for 10–27 households at each monthly visit (Fig. 1); however, no statistically significant difference was found between the included and excluded households in terms of socio-economic and demographic variables collected at baseline (data not shown). There were no significant differences in the distribution of HFI indices at the prior visit between the included and excluded households (all *p* > 0.05, Fig. 1). This study population had a high literacy rate (84.7%) among household heads (Table 1). About a quarter (24.5%) of household heads primarily worked in farming, while 20.7, 27.7 and 27.1%. of them were salaried workers, self-employed or in other occupations, respectively.

**Table 1:** Baseline characteristics of households the study population (*N* = 157)

Characteristics	N^a^ or Mean	% or (SD)
Characteristics of the households’ heads		
Literacy	127	84.7
Occupation		
Farming	38	24.5
Salaried employment	32	20.7
Self-employed/small business	43	27.7
Other	42	27.1
Characteristics of Households		
Religion		
Christian or equivalent^b^	133	85.3
Other	22	14.1
None	1	0.6
Tribe affiliation		
Lala	60	38.2
Bemba	38	24.2
Other	59	37.6
Primary language		
Lala	42	26.8
Bemba	93	59.2
Other	22	14.0

*Abbreviations*: *SD* standard deviation

^a^Differences between the total *N* and 157 reflect missing values

^b^Catholic, Seventh Day Adventist, Anglican, Jehovah’s Witness/Watchtower, Baptist, United Church of Zambia and Pentecostal were categorized as Christian or equivalent

**Fig. 2:**
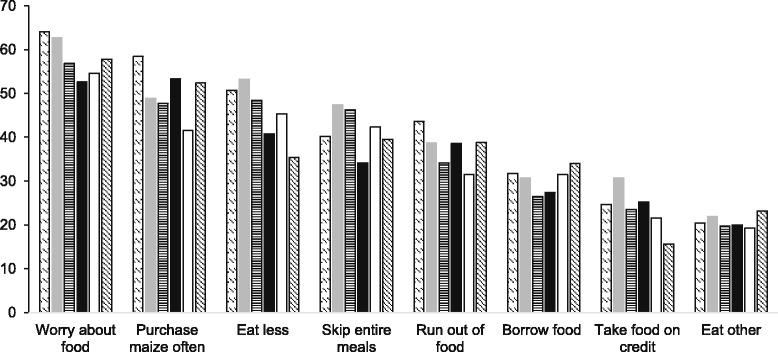
Percentage of affirmative responses (non-zero) to each item in the household food insecurity module at each monthly visit. Ranked by the averaged proportion across six visits. , visit 1; , visit 2; , visit 3; ■, visit 4; □, visit 5; , visit 6

The percentage of non-zero responses to each item of the HFI module is displayed by visit in Fig. 2. On average, the highest proportion of affirmative responses was observed in the “worry about food” item (52.6 to 64.1%) and the lowest proportion for the “eat other grains” item (19.2 to 23.1%). Across visits, the largest variability in the proportion of positive responses—calculated as difference between maximum and minimum percentage among visits—was 17.9%, in the “eat less” item (53.3% at visit 2 versus 35.4% at visit 6). The smallest variability was 3.9% in the “eat other grains” item (19.2% at visit 5 versus 23.1% at visit 6).

**Fig. 3:**
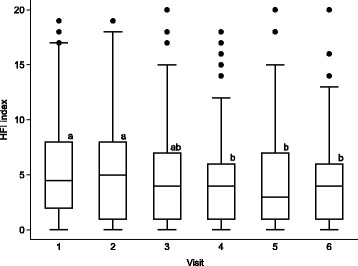
Distribution of HFI index by visit. Boxes represent median (middle line) and interquartile range. Whiskers represent upper and lower adjacent values. Dots represent outliers. **a**, **b** Distributions with different letters were different by the Wilcoxon matched signed-ranks test, *p* < 0.05. HFI, household food insecurity

Of a possible range of the HFI index from 0–32, we observed the index ranged from 0 to 20 in our sample (Fig. 3). The number (%) of households that reported a zero HFI index was 18 (12.7), 25 (18.3), 26 (19.7), 24 (17.8), 32 (24.6) and 26 (17.7%) at each visit, respectively. The distribution of HFI index was non-normal and the median [inter-quartile range, IQR] was 4.5 [2, 8], 5 [1, 8], 4 [1, 7], 4 [1, 6], 3 [1, 7] and 4 [1, 6], respectively. The HFI index at visits 1 and 2 was significantly higher than that at visits 4, 5 and 6 (all *p* < 0.05 in Wilcoxon matched signed rank test). However, distribution of HFI index by visit was not statistically different in all other pair-wise comparisons (*p* > 0.1, Fig. 3). Additional analysis comparing HFI patterns in households with heads primarily occupied by farming versus salary employment has revealed similar trends (data not shown).

**Fig. 4:**
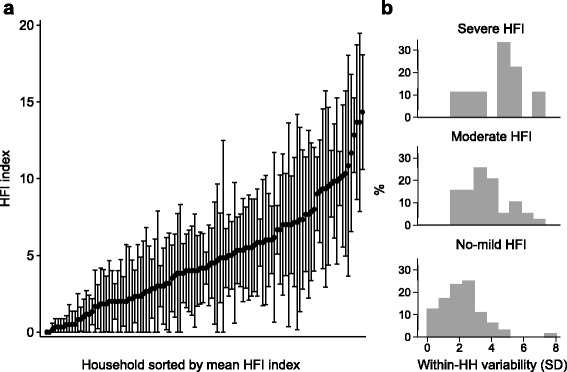
Mean and 95% confidence interval of HFI index over the six monthly visits by household **a** and distribution of the within-household variability of the HFI index (SD) by food insecurity group **b**. In panel *a*, for a given household, each dot is the mean value of index and the caps represent upper and lower bounds of the 95% confidence interval. Lower bounds are constrained to zero if negative. HFI, household food insecurity; SD, standard deviation

For consistent comparisons, only households with complete information in all six visits (*n* = 112) were plotted in the distribution of within-household food insecurity status over time (Fig. 4a). The range of mean index was 0–14.3. The proportion of households classified as having no-mild, moderate, and severe HFI was 57.1 (*n* = 64), 34.8 (*n* = 39), and 8.0 (*n* = 9), respectively. The ranges of 95% CI varied largely. As shown in Fig. 4b, we observed an increasing trend of the index's within-household variability in terms of standard deviation (median [IQR]) from no-mild (2.2 [1.2, 2.8]), moderate (3.4 [2.5, 4.4]) to the severe HFI group (4.8 [3.6, 5.1]). The Kendall’s correlation coefficients of HFI index among visit pairs ranged from 0.30 (between visit 1 and 6, visit 2 and 5) to 0.45 (visit 3 and 4) and all correlation coefficients were significant at 0.05 level. On average, there was a 0.25 point decrease in HFI index per visit (Table 2). Between- and within-household variance in HFI index was 8.8 and 10.7, respectively. The interclass correlation coefficient was 0.45.

**Table 2:** Estimates and variance structure from the random intercept model^a^

	Estimate	(95%CI)
Mean model		
Visit (β_1_)	−0.25	(−0.38, −0.12)
Constant (β_0_)	5.82	(5.12, 6.52)
Variance structure		
Between households (τ^2^)	8.81	(6.67, 11.64)
Within household (σ^2^)	10.66	(9.58, 11.87)
Interclass correlation coefficient (ρ)	0.45	

*Abbreviation*: *CI* confidence interval

^a^In the random intercept model, the HFI index is modeled as a linear function of visit clustered at household level. The model allows missing information in the follow-up assessments, therefore, the observations used in the analysis varied from 1 to 6 per household and averaged at 5.3 per household

## Discussion

In this rural Zambian setting, we found a low level of reported HFI and a minor decline in HFI status when assessed repeatedly over a six-month period.

The finding on declining average HFI status over time, though small in magnitude, was contrary to our original hypothesis. Apparent seasonal change in reported food insecurity was previously documented in Sub-Saharan Africa. For example, food insecurity both in Burkina Faso [14] and Tanzania [15] displayed a strong seasonal pattern with mean food insecurity score highest in the lean season and significantly lower in the post-harvest season. We therefore expected a rise, instead of a decrease, in food insecurity score (indicative of greater food insecurity) from post-harvest season (October) to the peak lean season (March) in rural Zambia.

A number of contextual factors may have explained the observed low level of HFI. Data on rainfall performance and cereal production suggested that there was no substantial shift in the food availability in Zambia from 2012 to 2013 [19]. The national retail price of maize increased by 32% from October 2012 to February 2013. As this was an expected seasonal fluctuation [20], it was unlikely to have had a significant impact on maize supply in the domestic market. Data from the United Nations further indicate that no urgent food aid was requested by the Zambian government during this period [21]. Although these measures were all at the national level and could not be tested due to lack of local-level data, stable food availability, anticipated price fluctuations, and limited food aid requirements likely held true in our study area. Considering the ecological factors, food insecurity status in rural Zambia likely remained at a low level over the six months of surveyed period. However, the seasonal price change implies food insecurity cycles. It is possible that households may have anticipated the upcoming lean season in March and April and thus employed strategies to reduce their food insecurity risk [22]. This may help explain the slight (0.25 point) decrease in reported food insecurity during a single lean season. Our results indicated that seasonal HFI assessments may be sufficient for periodic monitoring of food insecurity prevalence during non-crisis times at population level.

Interestingly, we found that reported food insecurity varied more within the same household over time than across households in our sample. The low correlation among repeated indices suggested challenges in predicting future measures of food insecurity solely based on the past reported status. High intra-household variation and low correlation implied the possibilities of both true variability in food insecurity status and measurement errors in the perception-based scale. Temporary food stress happens when unexpected shocks threaten the household's ability to produce or exchange for food. These may include the loss of household entitlements (e.g. agricultural resources, labor power or assets) or sudden changes in the relative price of foods or in food policy [23]. We anticipate that households with more severe food insecurity status (higher mean index) would be more vulnerable to short-term stresses (higher within-household variability). In our sample, we did observe a positive correlation between the household mean and standard deviation of the HFI index over the six-month period. Our findings suggest that within-household variability across repeated perception-based indices may help to identify households that are more susceptible to food stress, even if averaged status does not vary over time. Repeated assessments should be considered to avoid potential misclassification of food insecurity at household level, which may lead to inaccurate targeting of food security interventions or to biased estimation of relationships linking food insecurity with health outcomes [24].

Caution is always needed when interpreting perception-based indices, because measurement errors, rather than true variation, may also drive changes in responses. Sources of errors include: observation bias, response shifts, violation of internal validity and unreliability of the scale. Observation bias could occur if respondents associate their response to the items with their eligibility to receive food aid [25]. In the present study, households were informed during the consent process that they would receive a food package at the end of the six-month period. Thus, knowledge that the receipt of this package was independent of their survey responses should have minimized observation bias. Response shift refers to changes in perceptions over time due to adjustment of the respondent’s internal standards of food insecurity [25]. These may result from comparing current perceived food insecurity with past experiences, evaluating status in relation to neighbors, and/or using a mix of standards. Households that switched internal standards frequently across the monthly assessments would more likely have inconsistencies in their responses. Internal validity is supported if individual items represent “symptoms” of unobservable HFI. We have previously reported the internal validity of the FAST scale in this population [18]. However, variability observed in percent of affirmative responses indicate instability of performance of certain items in repeated measures. Scale reliability or dependability is based on the premise that changes in perceptions of food insecurity are in line with changes in objective measures [26]. The reliability of the FAST scale has been tested in Bangladesh using three rounds of data collected between 2000 and 2003 [17]. This study indicated that changes in the FAST index generally corresponded to changes in common comparators of food insecurity, including measures of consumption, asset holdings, and dietary diversity. However, the reliability of similar scales was inconsistent when examining the dynamic of reported food insecurity status before and after the 2008 food crisis [27, 28]. One study over this time period reported consistent relationships between HFI and both marked increases in food prices and decreases in household dietary diversity based on repeated cross-sectional surveys [27]. However, a separate prospective cohort study observed improvements in perceived food security that could not be fully explained by changes in household socio-economic status or food production measures, calling into question the inter-temporal validity of the HFI scale [28]. Scale reliability is seldom tested within a season or a calendar year. Our finding on high intra-household variation calls for future investigations on the degree of measurement error and inter-temporal validity of repeated perception-based measurements.

This study had several limitations. The six-month study period covered the lean season, so we were unable to extrapolate to HFI over the harvest- and post-harvest seasons. Furthermore, the present analysis did not include objective measures such as household-level dietary diversity as support for the latent HFI index. Though missing data was unlikely to be problematic in our study in terms of sample representativeness, our sample was purposively limited to households with 4- to 8-year old children. Households with and without children have been found to report different symptoms of food hardship [29], which may be due to differences in responsibility and priorities of maintaining food security. Thus, the generalizability of our findings may be limited to households with children in similar settings.

## Conclusions

In conclusion, we have provided longitudinal evidence of a slight decreasing trend in reported household food insecurity over a six-month period in the lean season in rural Zambia. The monthly perception measures of food insecurity demonstrated larger variation within household over time than variation across households. To understand the potential bias and misclassification, future research is needed to compare repeated perception-based food insecurity metrics with objective assessment of food availability and utilization at household- and individual-levels.

## Abbreviations

FAST: Food Access Survey Tool
HFI: Household food insecurity
